# Case Report: Coexistence of anti-LGI1 and anti-mGluR2 antibodies in an autoimmune encephalitis patient

**DOI:** 10.3389/fimmu.2025.1609389

**Published:** 2025-05-30

**Authors:** Xudong Zhang, Fei Ma, Qingqing Geng, Changjiang Luo, Chuanqiang Qu

**Affiliations:** ^1^ Department of Neurology, Shandong Provincial Hospital Affiliated to Shandong First Medical University, Jinan, China; ^2^ Department of Neurology, Jinan Shizhong District People’s Hospital, Jinan, China

**Keywords:** autoimmune encephalitis, LGI1 antibody, mGluR2 antibody, double antibody, acute cerebral infarction

## Abstract

Autoimmune encephalitis (AE) encompasses a broad group of inflammatory encephalopathies mediated by immune responses against central nervous system (CNS) antigens. With the expanding spectrum of identified anti-neuronal antibodies and their increasing clinical recognition, the number of confirmed AE cases has risen. Notably, cases involving concurrent positivity for multiple anti-neuronal antibodies have emerged, complicating both diagnosis and treatment. To date, no published reports have described the co-occurrence of anti-leucine-rich glioma-inactivated 1 (LGI1) antibody and anti-metabotropic glutamate receptor 2 (mGluR2) antibody in AE patients. We report a case of a 61-year-old woman presenting with impaired responsiveness, gait disturbance, and language disorders. Serological and cerebrospinal fluid (CSF) analyses revealed positivity for both LGI1 and mGluR2 antibodies. The anti-LGI1 antibody titers were 1:32+ (serum) and 1:1+ (CSF), while anti-mGluR2 antibody titers were 1:100+ (serum) and 1:10+ (CSF). Based on clinical manifestations and diagnostic findings, the patient was diagnosed with AE with concurrent anti-LGI1 and anti-mGluR2 antibody positivity. The patient received intravenous immunoglobulin (IVIG) and methylprednisolone pulse therapy (500 mg/day), resulting in symptomatic improvement. Following discharge, maintenance therapy with oral prednisone acetate and mycophenolate mofetil was initiated. At the one-week follow-up, her condition remained stable; however, she succumbed to death at the two-week follow-up due to complications from poor oral intake.

## 1 Introduction

Autoimmune encephalitis (AE), accounting for approximately 10-20% of encephalitis cases, represents a group of inflammatory brain disorders mediated by aberrant immune responses against central nervous system antigens (1). The clinical spectrum of AE is highly variable, encompassing seizures, cognitive dysfunction, psychiatric symptoms, and movement disorders (2). Based on the subcellular localization of target antigens, AE can be classified into two main categories: those associated with anti-neuronal intracellular antibodies and those with neuronal surface antibodies. The clinical presentation varies significantly depending on etiology, anatomical involvement, and the presence of paraneoplastic syndromes. With the expanding repertoire of identified anti-neuronal antibodies and improved diagnostic capabilities, an increasing number of AE cases with multiple antibody positivity have been recognized. Approximately 1.88% of patients with AE-related antibodies demonstrate this overlapping seropositivity, which adds considerable complexity to clinical evaluation and management. In this study, we present a rare case of AE with concurrent anti-LGI1 and anti-mGluR2 antibody positivity. By analyzing the clinical manifestations, auxiliary examinations, diagnostic process, and therapeutic response of this case in detail, we aim to accumulate experience in the diagnosis and treatment of the rare double-antibody-positive AE, and to improve the clinicians’ understanding of the rare antibodies and overlapping-antibody-associated AE.

## 2 Case presentation

**Figure 1:**
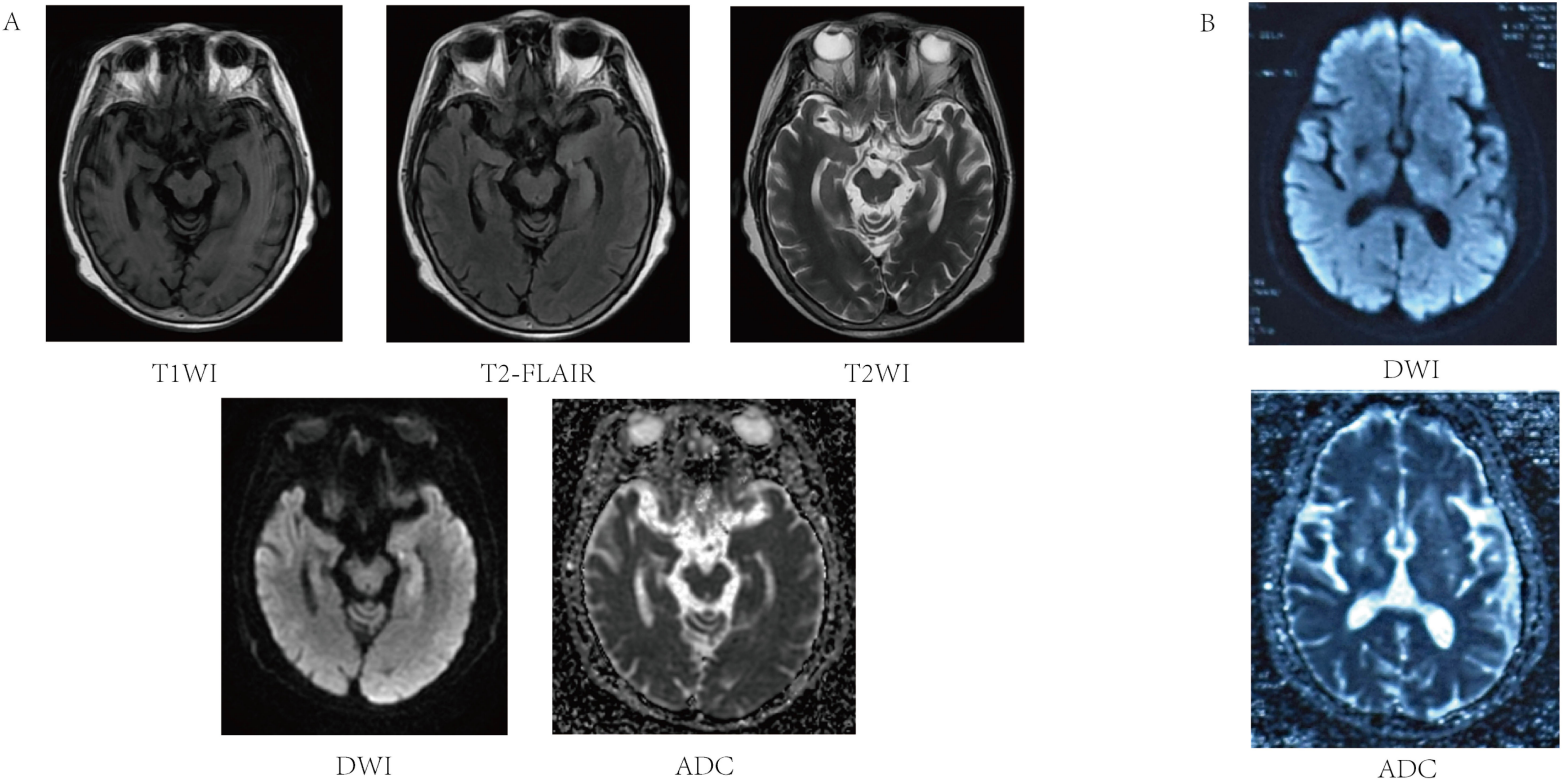
Magnetic resonance image of the patient’s acute cerebral infarction and left hippocampal abnormality. **(A)** T1 left hippocampus with reduced signal in T1WI sequence, slightly higher signal in T2-FLAIR, slightly higher signal in T2WI sequence increased signal in DWI sequence, and iso-signal on ADC maps. **(B)** Acute ischemic lesion in the left thalamus showing characteristic DWI hyperintensity and ADC hypointensity.

A 61-year-old female patient was admitted on 30 August 2024 due to impaired responsiveness, gait disturbance, and language disorders for 4 days, with symptom exacerbation in the preceding 48 hours. Four days before admission, her family noted unresponsiveness, abnormal behavior, difficulty in walking, slurred speech, and few words. Initial cranial MRI at a local hospital revealed an abnormal signal in the left hippocampus (**Figure 1A**) and acute infarction of the left thalamus (**Figure 1B**). The electrolytes showed a mild decrease in blood sodium (127.8 mmol/L, normal range: 135–145 mmol/L). After admission, the patient developed a fever, with a maximum body temperature of 38.6°C. The patient was diagnosed with ‘acute cerebral infarction’, and was given treatment to improve the circulation for 2 days. The patient’s above mentioned symptoms were aggravated, and she showed obvious impaired responsiveness, with occasional mild irritability, and she did not take the initiative to eat or drink water, and needed the assistance of her family members. She had difficulty in walking and needed help from others. For further diagnosis and treatment, she came to our hospital.

The patient’s past medical history included cerebral infarction and hypertension for over ten years, with residual effects including mild cognitive slowing and left facial paralysis. The patient had not been taking any regular medications for these conditions, and no other chronic diseases were reported. After admission, she was found to be alert but poorly responsive, with a depressed mood, cognitive decline, slurred speech, impaired expression, and only able to complete brief communication. The patient had no seizures, no headache, and no vomiting. Physical examination revealed muscle strength of both lower limbs at grade 4, negative meningeal signs, and she was uncooperative with the rest of the neurological investigations. Repeat cranial MRI confirmed persistent hippocampal signal abnormalities, suggestive of inflammatory etiology (**Figure 1A**). The patient’s electroencephalogram (EEG) was mildly abnormal, showing slowing of the background rhythm and enhancement of slow waves in the theta band. Combined with the clinical manifestations of recurrent fever and hyponatremia, the above features were inconsistent with typical cerebral infarction. Metabolic encephalopathy due to hyponatremia was ruled out first, and further examinations such as lumbar puncture and ultrasound were performed to detect autoimmune encephalitis.

**Figure 2:**
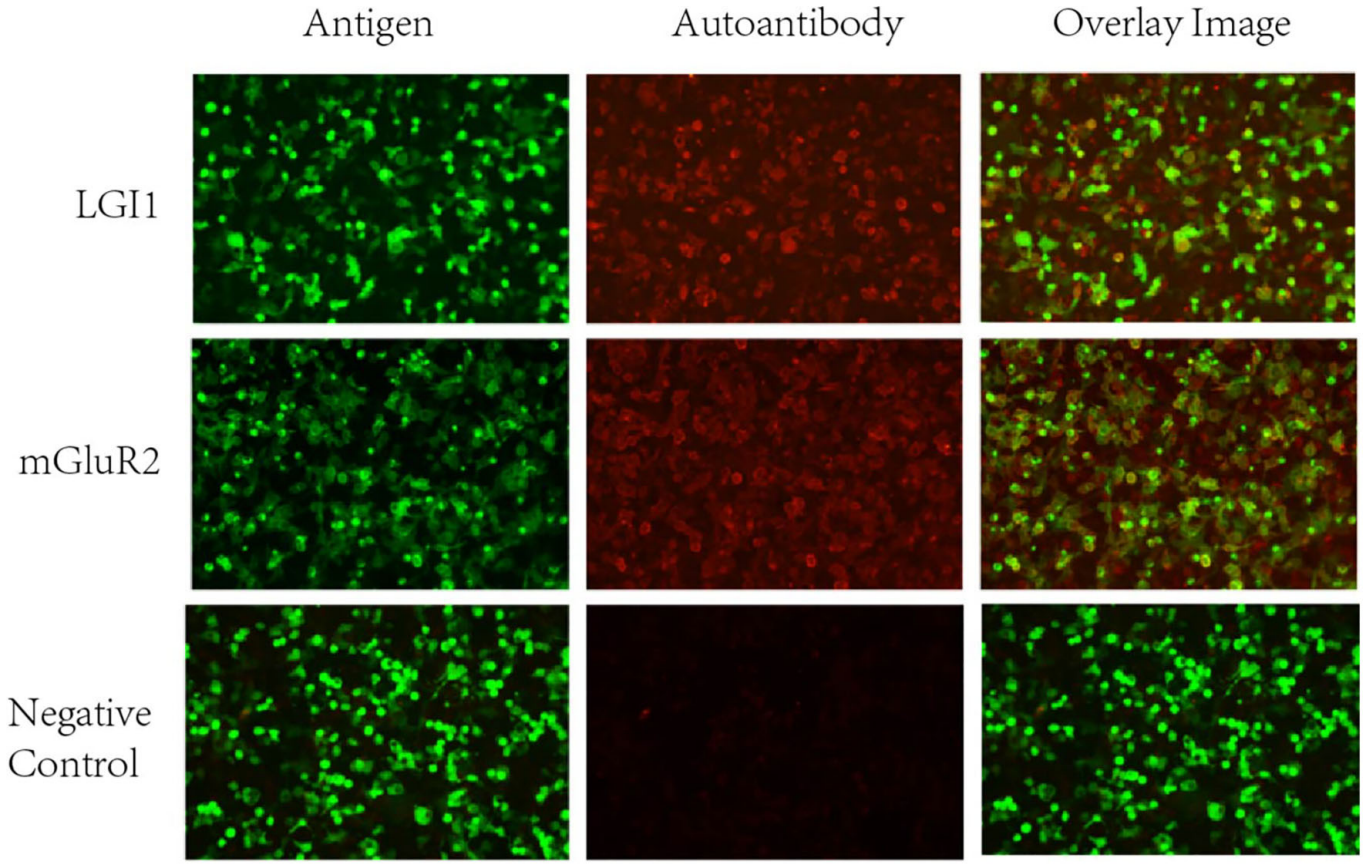
Cell Based Assay (CBA) showing the binding of serum anti-LGI1, mGluR2 antibodies in patients with autoimmune encephalitis (200x). It can be seen that patients’ serum anti-LGI1 and mGluR2 antibodies were bound to LGI1 and mGluR2 proteins present in HEK293T cells. Green: transfected cells (plasmid containing mEGFP). Red: autoantibodies.

Lower extremity arterial and venous vascular ultrasound suggested bilateral lower extremity atherosclerotic plaque formation, bilateral small saphenous vein old thrombophlebitis, and bilateral calf interosseous vein thrombosis. Abdominal ultrasound suggested bilateral renal cysts. Echocardiography indicated left ventricular dysfunction (LVEF: 56%), segmental wall motion abnormalities, mild valvular regurgitation (aortic, mitral, tricuspid), and pericardial effusion. Laboratory results indicated: hypokalemia (2.81 mmol/L, normal range: 3.5-5.5 mmol/L); high-sensitivity C-reactive protein (hs-CRP), 13.12 mg/L (normal range: 0–10 mg/L); serum amyloid A, 92.84 mg/L (normal range: 0–10 mg/L); D-dimer, 3.91 mg/L (normal range: 0-0.55 mg/L); N-terminal pro-brain natriuretic peptide (NT-proBNP), 1019.30 pg/mL (normal range<125 pg/mL). Lumbar puncture yielded clear, colorless CSF with a pressure of 118 mmH_2_O (normal range: 80–180 mmH_2_O). Routine examination of CSF suggested single nucleated cells of 26.00 x10^6^/L, multiple nucleated cells of 1.00 x10^6^/L, positive Pandy’s test (1+), a nucleated cell count of 27.00 x10^6^/L (normal range: 0-8.00 x 10^6^/L), and a protein quantification of 1.5 g/L (normal range: 0.15-0.45 g/L). Based on the patient’s medical history and comprehensive diagnostic evaluations, encephalitis could not be excluded from the differential diagnosis. Neural antibodies associated with autoimmune encephalitis including NMDAR, LGI1, GABABR, CASPR2, AMPAR1, AMPAR2, DPPX, IgLON5, GAD65, GlyR, mGluR5, D2R, MOG, GFAP, mGluR1, mGluR2, GABAARα1, GABAARβ3, NMDAR GluN2A, NMDAR GluN2B, Neurexin-3α, KLHL11, Cavα2δ, PDE10A, GluK2, AK5, KCTD16, NCAM1 in serum and CSF were tested using cell-based assays (CBA) with immunofluorescence double staining. The immunological testing revealed dual positivity for both anti-LGI1 and anti-mGluR2 antibodies. Specifically, the anti-LGI1 antibody demonstrated a serum titer of 1:32+ with a CSF titer of 1:1+, while the anti-mGluR2 antibody showed a serum titer of 1:100+ accompanied by a CSF titer of 1:10+ (**Figure 2**). Serum and CSF analysis for tissue-based assays (TBA) yielded negative results. All the detections were carried out in Jiangsu Simcere Diagnostic Laboratory (Jiangsu Simcere Diagnostics Co, Ltd, Nanjing 210002, China).

**Figure 3:**
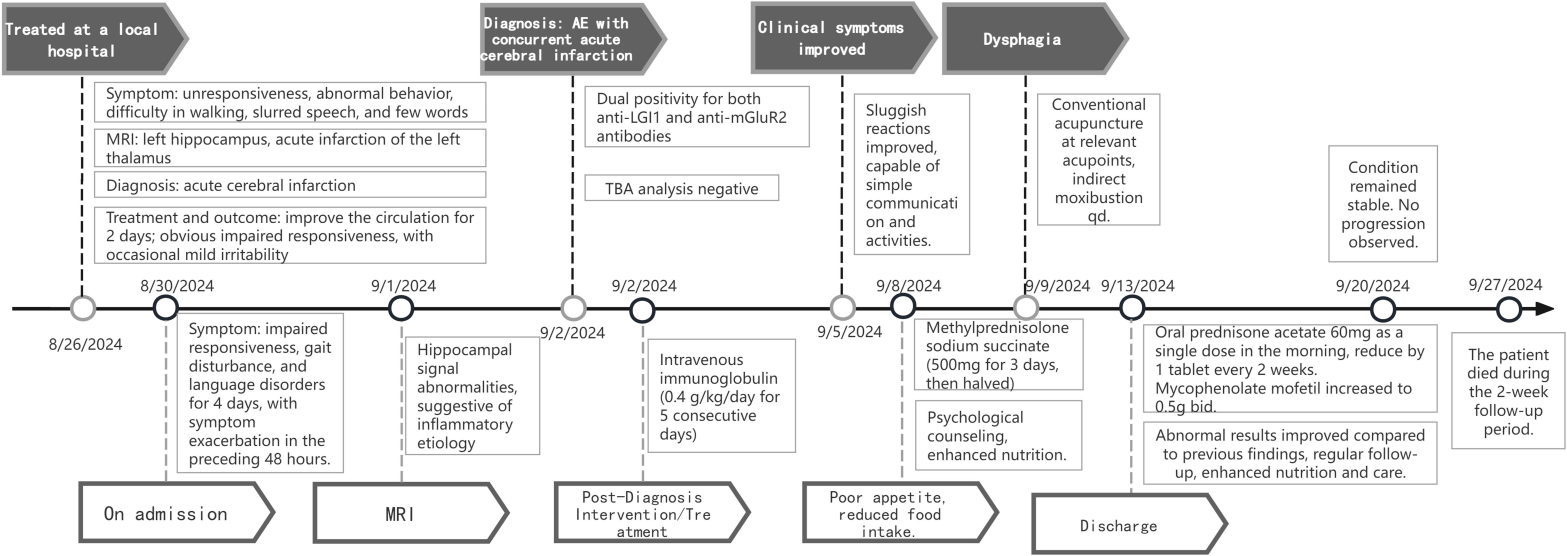
Timeline of the patient with clinical symptoms, diagnosis, and data before and after interventions. MRI, Magnetic Resonance Imaging; TBA, analysis for tissue-based assays; qd, every day; bid, twice a day.

We refined the patient’s tumor screening including routine screening for female tumor markers, ultrasonography (liver, biliary, pancreas, spleen, kidney etc.), and Computed Tomography (CT) of the chest. All results were negative, which ruled out the possibility of paraneoplastic syndrome. Herpes simplex virus nucleic acid test and TORCH (Toxoplasma, Others, Rubella, Cytomegalovirus, Herpes simplex virus) screen were negative, which helped to differentiate it from viral encephalitis, especially from herpes simplex virus encephalitis. The patient’s cerebrospinal fluid was negative for acid-fast staining and India ink staining, and this finding combined with the clinical presentation ruled out cryptococcal meningitis and tuberculous meningitis. Based on the clinical features, imaging findings, laboratory test results, and autoimmune encephalitis antibody detection, the patient was finally diagnosed with autoimmune encephalitis. Therapeutic interventions included subcutaneous administration of low molecular weight heparin for lower limb venous thrombosis, spironolactone for cardiac preload reduction with strict fluid intake monitoring, and immunomodulatory therapy consisting of intravenous immunoglobulin (0.4 g/kg/day for 5 consecutive days) combined with methylprednisolone sodium succinate pulse therapy (500 mg/day for 3 days). Following clinical improvement, the steroid regimen was transitioned to oral prednisone acetate (60 mg once daily in the morning) after a 3-day course of reduced-dose methylprednisolone (120 mg). Adjuvant mycophenolate mofetil (0.5 g twice daily) was concurrently initiated. Post-discharge follow-up at 1 week revealed no disease progression; however, the patient died due to poor feeding during the 2-week follow-up period The timeline of the patient, along with data on symptom improvement and detailed interventions, is shown in **Figure 3**.

## 3 Discussion

The patient presented with a constellation of neurological symptoms including cognitive impairment, psychiatric disturbances, unresponsiveness, and language disorders. Comprehensive diagnostic evaluations confirmed AE based on the detection of both LGI1 and mGluR2 antibodies in paired serum and cerebrospinal fluid samples. Notably, the paraneoplastic evaluation revealed no underlying malignancies. After combining the results of auxiliary examinations, the final diagnosis of AE was made, and the serum and cerebrospinal fluid were positive for anti-LGI1 and anti-mGluR2 antibodies, and the screening of the tumor was negative. To our knowledge, this is the first case of AE in which both anti-LGI1 and anti-mGluR2 antibodies were found to be positive in serum and CSF with negative tumor screening.

Lai et al. first reported anti-LGI1 antibody-associated encephalitis in 2010 and identified anti-LGI1 as the causative antibody (3). As the second most prevalent AE subtype following anti-N-methyl-D-aspartate receptor (NMDAR) encephalitis, anti-LGI1 encephalitis accounts for approximately 30% of limbic encephalitis cases. Typical clinical features of anti-LGI1 encephalitis include frequent epileptic seizures, mainly faciobrachial dystonic seizures (FBDS), cognitive decline, psychiatric-behavioral abnormalities, and autonomic dysfunction such as hyponatremia (4). LGI1 is a neuronal secretory protein that plays a key role in regulating neuronal excitability by forming a complex with presynaptic proteins ADAM11, ADAM22, and ADAM23 (5). Anti-LGI1 antibodies disrupt neurotransmission by blocking LGI1 binding to target proteins, leading to cognitive and behavioral deficits. Studies have shown that cognitive impairment in patients with anti-LGI1 AE may be caused by structural damage to the hippocampal memory system (6). Cognitive impairment, as one of the common neurological disorders in anti-LGI1 AE, increases the difficulty of differential diagnosis from neurodegenerative diseases. While initial presentation typically involves FBDS followed by encephalopathy, anti-LGI1 encephalitis is often misdiagnosed as viral encephalitis, psychiatric disorders, and stroke, leading to delayed diagnosis and treatment, and increasing patient morbidity and mortality.

Evidence suggests that immune-mediated pathogenesis underlies LGI1-associated rapidly progressive dementia characterized by predominant memory deficits (6). In addition, hyponatremia, a characteristic manifestation of anti-LGI1 AE, is considered to be a prodromal symptom and may be related to the syndrome of inappropriate secretion of antidiuretic hormone caused by simultaneous expression of LGI1 in the hypothalamus and kidneys (6). This pathophysiological correlation aligns with the observed electrolyte disturbance in our case. Current therapeutic guidelines recommend first-line immunotherapies including IVIG and high-dose corticosteroids for anti-LGI1 AE management (7). In the present case, the patient’s symptoms improved with intravenous immunoglobulin after the diagnosis of AE. When response to first-line treatment is suboptimal, Rituximab as second-line therapy not only demonstrates significant efficacy, but early administration of Rituximab can also effectively reduce the recurrence rate of AE (8–10).

Anti-mGluR2 is a G protein-coupled receptor mainly expressed in cerebellar, hippocampal, and cortical neurons, which plays an important role in regulating neurotransmitter release and neuronal excitability (11). mGluRs are widely involved in the modulation of a variety of physiological functions in the central and peripheral nervous systems, such as pain perception, anxiety, and learning and memory. These receptors are distributed in the presynaptic and postsynaptic membranes of neurons in the cerebellum, hippocampus, striatum, cerebral cortex, and other brain regions, as well as in the peripheral nervous system. According to the coupled intracellular signal transduction pathway and pharmacological properties, mGluRs can be classified into three groups of eight subtypes: group I includes mGluR1 and mGluR5, which are coupled with phospholipase C to activate the inositol triphosphate pathway and promote the release of intracellular calcium ions, while group II (mGluR2/3) and group III (mGluR4, 6-8) inhibit adenylate cyclase, negatively regulating cAMP-dependent signal transduction (12). Each mGluR isoform exists as a homodimer, and after binding and activation by two glutamate molecules, it plays important functions in the regulation of synaptic transmission, integration, and plasticity, and is involved in mediating slow excitatory and inhibitory postsynaptic potentials (12). Different subtypes are distributed in specific brain regions and neuron types and exercise different physiological regulatory roles.

Three types of mGluR encephalitis have been identified, namely anti-mGluR1, anti-mGluR2, and anti-mGluR5 encephalitis. Up to now, only four cases of anti-mGluR2 encephalitis have been reported (11, 13, 14), all of which were females presenting with cerebellar ataxia with dysarthria as the main clinical manifestation. Among these cases, two patients were found to have tumors, and three patients showed cerebellar abnormalities on head MRI. **Table 1** presents a comprehensive comparison of the clinical manifestations and relevant data across all five patients. In this case, the MRI of the head of a middle-aged woman revealed multiple lacunar infarcts and soft foci in the basal ganglia region and radiocorona bilaterally, acute infarction in the left thalamus, and abnormal signals in the left hippocampus. The left hippocampal signal abnormality in our patient is consistent with limbic encephalitis, a common manifestation of various forms of AE (15). However, the patient’s concomitant presentation of acute infarction of the left thalamus represents a novel finding. Thalamic involvement in autoimmune encephalitis is rare, with only one case reported in the literature, and no specific antibody positivity was detected in that case (16). We speculate that the acute infarction of the left thalamus may be associated with anti-mGluR2 antibodies, though the underlying pathophysiological mechanisms require further investigation.

**Table 1:** Comparison of characteristics among AE patients with anti-mGluR2-positive antibodies.

Case	Age, sex	Clinical presentation	MRI findings	Tumor association	Treatment	Outcome	Reference
1	56, female	Progressive ataxia, gait instability, dysarthria	A cerebellar ataxia and a Hot Cross Buns (HCB) sign	No malignancies identified	Intravenous glucocorticoids (1 g/day for 3 days, followed by 0.5 g/day for another 3 days)	She still had gait instability and dysarthria at follow-up on February 20, 2023.	(11)
2	32, female	Headache with memory decline for 14 days, abnormal mental behavior for 8 days, memory impairment, emotional blunting	Patchy abnormal signal adjacent to the right anterior horn and bilateral posterior horns of lateral ventricles	No potential tumor lesions identified	Methylprednisolone pulse therapy (starting dose of 1000 mg/day, halved every 5 days, then maintained at 80 mg/day intravenous infusion)	Significant improvement in consciousness state and psychiatric symptoms; clear consciousness, fluent speech, with psychomotor slowing	(13)
3	78, female	Progressive cerebellar ataxia, gait instability and dysarthria	Focal hyperintense cerebellar lesions, diffuse involvement of the cerebellar white matter	A small-cell tumor of unknown origin	Unresponsive to intravenous immunoglobulin or corticosteroids, with only partial response to one cycle of rituximab	Died a few months after tumor diagnosis	(14)
4	3, female	Gait instability, dysarthria, horizontal right-beating nystagmus, limb and truncal ataxia, and broad-based gait requiring bilateral support	Patchy gadolinium enhancement in the cerebellar folia	An alveolar rhabdomyosarcoma	Intravenous methylprednisolone and immunoglobulins	Full recovery	(14)
Curre-nt case	61, female	Impaired responsiveness, gait disturbance, and language disorders for 4 days	Acute cerebral infarction, left hippocampal abnormality	No potential tumor lesions identified	Intravenous immunoglobulin (0.4 g/kg/day for 5 consecutive days) combined with methylprednisolone sodium succinate pulse therapy (500 mg/day for 3 days)	Died at the two-week follow-up due to poor oral intake.	–

To date, there is only one reported international case of negative tumor screening in a patient with anti-mGluR2 antibody-associated AE (10). Extensive tumor screening in our patient also revealed no evidence of tumor. The reported cases of anti-mGluR2 antibody encephalitis were mainly characterized by psychiatric disorders, accompanied by headache and memory loss. In the present case, in addition to the above symptoms, behavioral and speech disorders were also observed, but both of them were characterized by unresponsiveness. It is assumed that the co-existence of the two antibodies may have led to the specific clinical phenotype. At present, there are very limited domestic and international case reports of AE associated with anti-mglur2 antibodies, and the specific pathogenic mechanism and clinical features are still unclear.

In the literature, anti-mGluR2 antibody-positive encephalitis is characterized by cerebellar ataxia, while anti-LGI1 antibody-positive encephalitis is characterized by limbic symptoms. In this case, the presence of both antibodies resulted in a variety of clinical manifestations, such as cognitive impairment, language and behavioral abnormalities, and unresponsiveness, which may be attributed to the synergistic effect of the two antibodies on different brain regions. There is a certain degree of correlation between the symptoms of AE and the type of antibody, and when different antibodies work together, they may present more complex and varied clinical features.

It is important to note that in patients with paraneoplastic syndromes, neurological symptoms may precede tumor diagnosis by several years. Therefore, even if the initial tumor screen is negative in patients diagnosed with AE, vigilance and regular follow-up are required to avoid missing the underlying tumor. In addition to known tumor types, other rare tumors should also be considered. Early diagnosis of AE is difficult due to the lack of specificity in its early clinical manifestations and ancillary investigations, and AE with psychiatric behavioral abnormalities as the first symptom can easily be misdiagnosed as psychiatric-related diseases and delayed treatment (5).

The current study has practical clinical significance: (1) Patients with behavioral abnormalities should be alerted to the possibility of AE, and lumbar puncture and autoantibody testing should be improved as early as possible. (2) AE may be the first manifestation of paraneoplastic syndrome, and patients should be examined for the presence of underlying tumors during clinical follow-up.

## 4 Conclusion

In conclusion, this case reports the first AE in which both anti-LGI1 and anti-mGluR2 antibodies were detected positively, providing new insights into a rare clinical phenotype in which the two antibodies work together. The contribution of anti-mGLuR2 and anti-LGI1 antibodies to the patient’s symptoms needs to be further explored. AE should be included in the differential diagnosis of acute and chronic cognitive dysfunction of unknown cause, and early recognition and treatment may improve the prognosis. Patients with encephalitis should also be alerted to the potential risk of tumors, and long-term follow-up is essential.

## Data Availability

The data analyzed in this study is subject to the following licenses/restrictions: To protect patient privacy and ethical limitations, the data sets provided in this study are not readily available. To access the data sets in this study please ask the appropriate authors. Requests to access these datasets should be directed to Chuanqiang Qu, drquchuanqiang@sina.com.
